# Changes in cognition of ADRD patients: effects of social isolation proxies, utilising data from UK electronic health records

**DOI:** 10.1002/alz.089448

**Published:** 2025-01-09

**Authors:** James A C Myers, Tom Stafford, Nemanja Vaci

**Affiliations:** ^1^ University of Sheffield, Sheffield United Kingdom

## Abstract

**Background:**

Marital status and living status are components of social isolation (SI), a modifiable factor thought to impact cognitive resilience, which has the potential to impact cognition throughout the course of Alzheimer’s and related dementia (ADRD) diagnosis. Electronic health records (EHRs) offer access to large scale clinical data, capable of longitudinal analyses.

**Method:**

Cognitive function measurement – Montreal Cognitive Assessment (MoCA) – data, demographic (including marital and living status as SI proxies) data and ADRD diagnosis data from patients aged 50+ years from Oxford Health NHS Foundation Trust (UK) were extracted using natural language processing algorithms from EHRs dated 1995 to 2022. Longitudinal multilevel models were used to predict cognition as a function of the interaction between diagnosis duration and SI proxies, controlling for age, sex and diagnosis cause.

**Result:**

‘Lifelong single’ marital status significantly predicted reduced cognition intercept scores for the MoCA dataset (𝛽 = ‐1.61, SE = 0.67, t = ‐2.42, p = 0.016). No significant marital status predictors for slope were found. Living in supported accommodation significantly predicted steeper slopes for cognition (𝛽 = ‐2.37, SE = 0.33, t = ‐7.20, p < 0.001). No living status levels significantly predicted slopes.

**Conclusion:**

Worldwide ADRD incidence is predicted to increase dramatically within the next 30 years, therefore studies investigating the impact of modifiable factors on the rates of cognitive change in ADRD patients are valuable to enhancing understanding of patient care. SI data extracted from EHRs can be used to predict differences in patient cognition scores.

